# Fatty Pancreas and Pancreatic Cancer: An Overlooked Association?

**DOI:** 10.3390/jcm11030763

**Published:** 2022-01-30

**Authors:** Tawfik Khoury, Wisam Sbeit

**Affiliations:** 1 Department of Gastroenterology, Galilee Medical Center, Nahariya 22100, Israel; wisams@gmc.gov.il; 2 Faculty of Medicine in the Galilee, Bar-Ilan University, Safed 5290002, Israel

**Keywords:** pancreas, fat, cancer, infiltration

## Abstract

Background: fatty pancreas (FP) is an old observation, but a new disease with clinical implications and several associated comorbid conditions, ranging from mild to life-threatening diseases. Herein, we aimed to assess the association between FP and pancreatic cancer (PC) development. Methods: we performed a retrospective cross-sectional study including all patients who underwent endoscopic ultrasound (EUS) for hepatobiliary indications. The study cohort was divided into patients with and without PC. Univariate and multivariate analysis were used to assess the association of several parameters with PC. Results: overall, 519 patients were included in the study. Of them, 48 had PC (PC group), and 471 did not (non-PC group). In univariate analysis, age (OR 1.04, 95% CI 1.01–1.07, *p* = 0.004), congestive heart failure (CHF) (OR 3.89, 95% CI 1.72–8.79, *p* = 0.001), ischemic heart disease (IHD) (OR 3.36, 95% CI 1.59–7.05, *p* = 0.001), hypertension (OR 2.42, 95% CI 1.33–4.41, *p* = 0.004) and fatty pancreas (FP) (OR 2.62, 95% CI 1.23–5.57, *p* = 0.01) were significantly associated with PC. In multivariate logistic regression analysis, only FP kept its association (OR 2.35, 95% CI 1.04–5.33, *p* = 0.04). Conclusion: FP was significantly associated with PC. A follow-up plan should be considered for individuals with FP.

## 1. Introduction

Fatty pancreas (FP) is defined by fat infiltration of the pancreatic parenchyma in the absence of significant alcohol consumption [1]. The prevalence of FP ranges from 16–35% according to several studies of abdominal ultrasonography imaging [2,3]. Previously, FP was thought to be a benign incidental finding in imaging studies performed for other causes and its clinical implications were not thoroughly investigated for several decades since it was initially reported in the early 1930s. However, in recent years, there is accumulating evidence supporting the association of FP with several comorbid diseases. Most importantly, it is associated with metabolic syndrome, obesity, diabetes mellitus [4] and with non-alcoholic fatty liver disease (NAFLD) [5]. Moreover, there are some conflicting data regarding its association with PC. Therefore, the aim of the present study was to assess the existence of this association between FP and PC.

## 2. Materials and Methods

We performed a retrospective cross-sectional study at Galilee Medical Center from 2015 through 2020, including all patients above 18 years old who underwent endoscopic ultrasound (EUS) for hepatobiliary indications. The study cohort was divided into two groups according to the presence of PC diagnosed morphologically and confirmed histologically. Patients’ files were accessed, and the extracted data included demographics, baseline characteristics, medical history and data regarding the presence or absence of FP and pancreatic PC. The study was approved by the local ethics committee and written informed consent was waived due to the retrospective non-interventional study design.

### 2.1. Diagnostic Criteria Used in Our Cohort

The diagnosis of FP was based on EUS, which is considered a routine imaging modality to visualize the pancreas [3,6]. The ultrasonographic appearance of FP is diffuse hyper-echogenicity compared to the adjacent solid organs, with comparisons often made with the kidneys [6]. PC diagnosis was based on morphological endosonographic appearance and definite histological diagnosis of carcinoma as reported by an experienced gastrointestinal pathologist.

### 2.2. Statistical Analysis

The study cohort was divided into patients with confirmed histopathological diagnosis of PC, compared to patients without. Then, descriptive analysis was conducted to compare the two groups. Continuous variables were reported as means ± standard deviation, and frequencies (percentages) for categorical variables. Wilcoxon signed-rank and Fisher’s exact probability tests were used where appropriate, and univariate analysis was used to estimate the significance of the assessed parameters by reporting odds ratio (OR) and confidence interval (CI), and multivariate logistic regression analysis was used to eliminate the effect of confounders and to find the final parameters associated with PC using logistic regression with a backward selection model, calculated by Fisher’s exact probability test. A threshold for statistical significance was set at a *p* value < 0.05. All analyses were performed by an experienced statistician using the statistical analysis software (SAS vs. 9.4 Copyright (c) 2016 by SAS Institute Inc., Cary, NC, USA).

## 3. Results

### 3.1. Demographics and Baseline Characteristics

Overall, 519 patients who had had EUS performed due to various hepato-pancreato-biliary indications were included in the study. Among them, 48 had PC (PC group), and 471 did not (non-PC group). The average age in the PC group was 68.7 ± 10.5, as compared to 62.5 ± 14.2 years in the non-PC group. Notably, male gender was significantly less common in the PC group (18 patients, 37.5%), vs. 265 patients (56.3%) in the non-PC group. There was no difference in the rates of smoking, obesity and diabetes mellitus among both groups. However, hypertension, ischemic heart diseases (IHD), hyperlipidemia and congestive heart failure (CHF) were more common in the PC group (45.8%, 22.9%, and 18.7%), vs. 25.9%, 8.3%, and 5.7% in the non-PC group, respectively. Interestingly, 20.8% of patients in the PC group had endosonographic evidence of FP, compared to only 9.3% in the non-PC group. Additionally, four patients with PC (8.3%) had distal pancreatic atrophy in the PC group, as compared to 14 patients (3%) in the non-PC group (*p* = 0.054) (Table 1).

### 3.2. Univariate and Multivariate Logistic Regression Analysis of Parameters That Were Associated with Pancreatic Adenocarcinoma

On univariate analysis, several parameters were significantly associated with PC, including age (OR 1.04, *p* = 0.004), CHF (OR 3.89, *p* = 0.001), IHD (OR 3.36, *p* = 0.001), hypertension (OR 2.42, *p* = 0.004) and FP (OR 2.62, *p* = 0.01), while male gender was negatively associated with PC (OR 0.47, *p* = 0.01) (Table 2). However, in multivariate logistic regression analysis incorporating all parameters that were significant on univariate analysis, we found that male gender kept its negative association with PC (OR 0.47, 95% CI 0.24–0.89, *p* = 0.02), while dyslipidemia kept its association (OR 2.43, 95% CI 1.06–5.54, *p* = 0.03) and FP remained significantly associated with PC (OR 2.35, 95% CI 1.04–5.33, *p* = 0.04) irrespective of dyslipidemia. Age showed a trend for significance with PC (OR 1.03, 95% CI 1–1.06, *p* = 0.05) (Table 3).

## 4. Discussion

FP was reported as early as the 1930s; however, for many decades it was perceived as a benign incidental finding, and therefore the clinical consequences of this condition were actually overlooked [1]. The last several years have witnessed accumulating evidence on the clinical implications of FP, including its association with non-cancerous conditions such as diabetes mellitus, metabolic syndrome and cardiovascular diseases [7], in addition to NAFLD [5]. Moreover, recent studies have shown that FP is a risk factor for acute pancreatitis [8], and have described an association with pre-cancerous pancreatic mucinous neoplasm [9]. On the other hand, the evidence supporting the association with pancreatic cancer is still limited. Herein, in our study we retrospectively evaluated risk factor for PC in a large cohort of patients who underwent EUS. We found that FP remained the only significant parameter associated with PC (OR 2.35, 95% CI 1.04–5.33, *p* = 0.04). Our results were in alignment with previous studies that reported a positive association. Lesmana et al. reported that FP was the only significant risk factor for pancreatic cancer in a regression analysis [10]. Moreover, further studies have shown that FP is directly correlated with the development of pancreatic intra-epithelial neoplasia and ductal adenocarcinoma [11,12]. Additionally, FP was shown to worsen the prognosis of PC, as it promotes pancreatic cancer dissemination [13]. The postulated pathogenesis behind this association is secondary to two patho-mechanisms; the first one is linked to obesity, which is associated with oxidative stress and adipokines imbalance, which leads to a low-grade chronic systemic inflammatory state that predisposes for recurrent pancreatitis, which is considered a major risk factor for pancreatic cancer [14]. The second one is related to the effect of intra-pancreatic fatty infiltration on the development of pancreatic cancer by inducing steato-pancreatitis leading to pancreatic cell injury, fibrosis and finally cancer [15,16]. Additionally, recent data have highlighted the role of gut microbiota in the development of PC and in tumor progression, as it was shown that the gut microbial diversity was lower among patients with PC, accompanied by an increment in potentially harmful pathogens such as Enterobacteriaceae and a decrease in some beneficial probiotics such as Bifidobacterium and butyrate-producing bacteria, suggesting that restoring the state of microbiota among PC patients might be associated with a favorable disease outcome [17].

In our study we found a trend for a higher rate for pancreatic atrophy in the PC group. This finding is expected, as this finding is secondary to the PC-associated obstructive pancreatitis. A recent study showed that pancreatic atrophy was present in approximately 34% of patients with PC, as even the authors suggested that pancreatic atrophy might be an early sign of the development of PC [18]. In our study, we did not have data regarding the presence of pancreatic atrophy prior to the diagnosis of PC. However, further studies are needed to precisely assess the consequences of pancreatic atrophy in the absence of other concomitant causes at the time of diagnosis.

Additionally, we found a significant correlation of dyslipidemia with PC. We assume that it is not a casual association, but it could be explained by its coexistence with FP [19]. Moreover, male gender was negatively associated with PC (0.47, 95% CI 0.24–0.89) in our cohort; this finding is contrary to the literature, as it was shown that PC is slightly more common in males than in females [20]. However, in our cohort, the average age in the PC group was significantly higher compared to the non-PC group; thus, the higher female-to-male ratio in the PC group might be explained by the fact that PC cases occurred at an older age in females compared to males, according to the Global Burden of Disease Study [21].

There are several risk factors for pancreatic cancer, including smoking [22], significant alcohol consumption [23], chronic pancreatitis [24,25], obesity [26], diabetes mellitus [27,28] and age [29]. In our study, there was no effect of smoking, obesity, chronic pancreatitis and diabetes mellitus, while only a trend for significance for age was found. Our findings further strengthen the role of FP in the development of PC. Notably, we were unable to extract data regarding alcohol consumption; however, given the fact that chronic alcohol consumption mainly causes chronic pancreatitis and that chronic pancreatitis prevalence in our cohort was low and similar between the two groups, the expected bias on the association of FP and PC from the unavailability of the data regarding alcohol consumption is marginal.

One limitation of our study is its retrospective single-center-study design. In addition, we did not have data regarding genetic mutation testing in this setting. However, as the average age in our cohort was relatively old, the bias from not having genetic testing is minor. On the other hand, the strengths are the relatively large number of patients included and that the diagnosis of FP was performed by EUS, which has very high sensitivity and specificity for the diagnosis of this condition.

In conclusion, FP was found to be a significant risk factor for PC. Given the accumulating evidence of this association, coupled with the fact that FP can be a reversible disease, proactively identifying individuals with FP is of paramount importance, as implementing a management plan, especially to patients with metabolic syndrome and obesity, might reduce pancreatic steatosis and minimize the risk of PC development. Moreover, clinicians should support and encourage individuals with FP to abandon other lifestyle factors causing pancreatic cancer, such as smoking and heavy alcohol drinking.

## Figures and Tables

**Table 1:** Demographics and baseline characteristics.

	PC Group	Non-PC Group	*p* Value
N	48	471	-
Age, mean ± SD (years)	68.7 ± 10.5	62.5 ± 14.2	0.02
Male gender, N (%)	18 (37.5)	265 (56.3)	0.01
Smoking, N (%)	6 (12.5)	45 (9.6)	0.51
Obesity, N (%)	9 (18.7)	94 (20)	0.84
Congestive heart failure, N (%)	9 (18.7)	27 (5.7)	0.007
Ischemic heart disease, N (%)	11 (22.9)	39 (8.3)	0.001
Diabetes mellitus, N (%)	13 (27.1)	85 (18.1)	0.13
Hypertension, N (%)	22 (45.8)	122 (25.9)	0.003
Dyslipidemia, N (%)	12 (25)	39 (8.3)	0.002
Chronic pancreatitis, N (%)	2 (4.2)	17 (3.6)	0.84
Pancreatic atrophy, N (%)	4 (8.3)	14 (3)	0.054
Fatty pancreas, N (%)	10 (20.8)	44 (9.3)	0.01

PC: Pancreatic cancer; N: Number.

**Table 2:** Univariate analysis of parameters associated with PC.

	OR	95% CI	*p* Value
Male gender	0.47	0.26–0.86	0.01
Age	1.04	1.01–1.07	0.004
Obesity	0.96	0.46–2.03	0.92
Congestive heart failure	3.89	1.72–8.79	0.001
Ischemic heart disease	3.36	1.59–7.05	0.001
Smoking	1.43	0.59–3.48	0.43
Diabetes mellitus	1.72	0.88–3.36	0.11
Hypertension	2.42	1.33–4.41	0.004
Dyslipidemia	3.75	1.81–7.75	0.004
Chronic pancreatitis	1.16	0.26–5.18	0.84
Fatty pancreas	2.62	1.23–5.57	0.01

OR: Odds ratio; CI: Confidence interval.

**Table 3:** Multivariate logistic regression analysis of parameters associated with PC.

	OR	95% CI	*p* Value
Male gender	0.47	0.24–0.89	0.02
Age	1.03	1–1.06	0.05
Congestive heart failure	1.65	0.65–4.22	0.29
Ischemic heart disease	1.37	0.56–3.38	0.49
Hypertension	1.12	0.54–2.35	0.76
Dyslipidemia	2.43	1.06–5.54	0.03
Fatty pancreas	2.35	1.04–5.33	0.04

## Data Availability

The data are available with the corresponding authors at the Gastroenterology department at Galilee Medical Center and will be available upon reasonable request.

## Abbreviations

FP: fatty pancreas; NAFLD: non-alcoholic fatty liver disease; PC: pancreatic cancer; EUS: endoscopic ultrasound; CHF: congestive heart failure; IHD: ischemic heart disease; FP: fatty pancreas; OR: odds ration; CI: confidence interval
